# Synergistic Effect of Fadrozole and Insulin-Like Growth Factor-I on Female-To-Male Sex Reversal and Body Weight of Broiler Chicks

**DOI:** 10.1371/journal.pone.0103570

**Published:** 2014-07-30

**Authors:** Mohammad Mohammadrezaei, Majid Toghyani, Abbasali Gheisari, Mehdi Toghyani, Shahin Eghbalsaied

**Affiliations:** 1 Young Researchers and Elite Club, Khorasgan (Isfahan) Branch, Islamic Azad University, Isfahan, Iran; 2 Department of Animal Science, Khorasgan (Isfahan) Branch, Islamic Azad University, Isfahan, Iran; 3 Department of Animal Science, Isfahan Research Center for Natural Resources and Agriculture, Isfahan, Iran; 4 Department of Animal Science, School of Environmental and Rural Science, University of New England, Armidale, New South Wales, Australia; Laboratoire de Biologie du Développement de Villefranche-sur-Mer, France

## Abstract

The aim of this study was to investigate the effects of Fadrozole hydrochloride and recombinant human insulin-like growth factor I (rhIGF-I) on female-to-male sex reversal, hatching traits, and body weight of broiler chickens. On the third day of incubation, fertile eggs were randomly assigned to five experimental groups comprising (i) Fadrozole (0.1 mg/egg), (ii) rhIGF-I (100 ng/egg), (iii) Fadrozole (0.1 mg/egg) + rhIGF-I (100 ng/egg), (iv) vehicle injection (10 mM acetic acid and 0.1% BSA), and (v) non-injected eggs. Eggs in the rhIGF-I-injected groups showed the mode of hatching time at the 480th hour of incubation, 12 hours earlier compared to the other groups, with no statistically significant difference in mortality and hatchability. On Day 1 and 42 of production, 90% of genetically female chicks were masculinized using Fadrozole treatment, while 100% female-to-male phenotypic sex reversal was observed in the Fadrozole+rhIGF-I group. Fadrozole equalized the body weight of both genders, although rhIGF-I was effective on the body weight of male chicks only. Interestingly, combined rhIGF-I and Fadrozole could increase the body weight in both sexes compared to the individual injections (P<0.05). These findings revealed that (i) IGF-I-treated chicken embryos were shown to be an effective option for overcoming the very long chicken deprivation period, (ii) the simultaneous treatment with Fadrozole and IGF-I could maximize the female-to-male sex reversal chance, (iii) the increase in the body weight of masculinized chickens via Fadrozole could be equal to their genetically male counterparts, and (iv) the IGF-I effectiveness, specifically along with the application of aromatase inhibitors in female chicks, indicates that estrogen synthesis could be a stumbling block for the IGF-I action mechanism in female embryos.

## Introduction

Sexual differentiation in the avian system is directed by the presence or absence of the W chromosome, similar to the Y chromosome in mammals [1]. Unlike mammals, genetically male birds are homozygous (ZZ) and the females are heterozygous (ZW) [2], [3]. It is thought that, like in mammals, one or both of the chicken sex chromosomes carry genes which control the cellular decision-making process for gonad development, resulting in ovary development in ZW hens and testis in ZZ roosters [4]. The onset of gonadal sex differentiation in birds is sensitive to steroid hormones [5]. Estrogen is required for ovarian development and control proliferation of the left gonadal cortex [6]. Moreover, 17β-Hydroxysteroid dehydrogenase and aromatase enzymes which are responsible for the conversion of androgens to estradiol-17 β are only detectable in the female embryos’ gonads [7]. Accordingly, estradiol can be detected from E9 to E15 in female chicken embryonic gonads by monitoring the production of estradiol-17β [8].

The importance of aromatase is underscored by numerous studies showing that aromatase inhibitors, such as Fadrozole, induce female-to-male sex reversal in ZW females when administered before or during sexual differentiation [9]–[11]. After a treatment on the third day of egg incubation with 1-methyl-androstendion, a steroidal aromatase inhibitor, or with Fadrozole, a nonsteroidal aromatase inhibitor, gonads of 12-day-old female embryos looked like testis and exhibited different grades of sex reversal [12]. In another study, the administration of Fadrozole prior to the incubation indicated that gonads of the majority of females had a visual appearance of testes at the hatching time [11]. Recently, Li et al. [13] showed that injection of Fadrozole before gonadal sex differentiation on the third day of incubation can induce female-to-male sex reversal in broiler chickens. On the other hand, the male-to-female chicken sex reversal can be directed by the addition of estrogen [6] or aromatase over-expression [1] during sexual differentiation, pointing out the linearity in the aromatase-estrogen pathway.

Insulin-like growth factors, IGF-1 and IGF-2, play essential roles in mammalian and avian growth and development as a mediator of growth hormone, either as endocrine or autocrine-paracrine effectors [14]. The positive impact of *in ovo* administration of IGF-I on embryonic and postnatal growth and development, particularly skeletal muscles, is well documented in broiler chicks and quails [15]–[17]. Although IGF-I has been fully documented as a growth hormone in various species, its interaction with aromatase/anti-aromatase has not been well explored. It has been reported that IGF-I plays an important role in the regulation of testicular steroid biosynthesis, testicular growth, and development [18]. However, the regulation of aromatase gene expression using IGF-I manipulation appears to be different between mammalian Sertoli and granulosa cells [19], [20].

Male broiler chicks have a faster growth rate and better feed efficiency than females and are thus of particular economic interest for farmers. Even though acceptable sex reversal rate has been achieved through aromatase inhibitors, female-to-male sex-reversed chicks do not gain much weight compared to the genetically male chickens [1], [11]. Treatment of chicken embryos by IGF-I, as an important growth inducer, could be effective for overcoming the lower weight gain drawback of sex-reversed chicks. However, IGF-I effectiveness on the body weight gain is exclusively limited to the male chicks [16], [21], [22], and it still needs to be investigated if inhibiting estrogen synthesis could trigger the IGF-I efficiency in genetically female chicks. In addition, the possible synergistic or antagonistic impacts of anti-aromatase and IGF-I on chicken sex reversal have not been studied. Thus, the objective of the current study was to investigate the effect of *in ovo* administration of Fadrozole and recombinant human (rh) IGF-I on female-to-male sex reversal and body weight of broiler chicks. Hatching characteristics were also investigated to detect possible detrimental treatment effects.

## Materials and Methods

### Ethical statements

On Day 4 of incubation, an *in ovo* injection was carried out over a large distance from the embryo location to minimize the physical damages done to the embryos. Eggs which did not hatch until Day 22 of incubation were cracked by the staff, and the dead embryos were used for genotyping. No mortality was observed during the production period in all treatment groups. In each measurement session, each chick was handled only for few minutes to avoid unnecessary stress and improve chicken refinements. By the end of Day 42 of production, chicks from all groups were promptly and humanely killed by a panel of experts and used for morphological evaluations. It should be borne in mind that the researchers of the current study had recruited two enthusiastic, well-trained, and skilled staff to thoroughly monitor the chicken’s behavior and physical appearance, as well as feed and water intakes throughout the production period. All the experimental procedures were assessed and approved by the Institutional Animal Care and Ethics Committee in Islamic Azad University, Khorasgan Branch, Isfahan, Iran, and all national and institutional guidelines were appropriately followed.

### Experimental treatments and injection procedure

A total number of 550 fertilized commercial eggs coming from the Ross broiler (308) chickens were collected and incubated under standard conditions (temperature 37.5±0.2°C, relative humidity 65±2%). On the third day of incubation, 85 hours following the eggs being placed in the incubator, all the set eggs were candled and the air sacks were located. Fertile eggs (65±1.5 g) were randomly divided into five groups with four replicates of 24 eggs; namely i) a Fadrozole hydrochloride-injected (#F3806, Sigma-Aldrich, Germany; 0.1 mg per egg) group, ii) a recombinant human (rh) IGF-I injected (#SRP3069, Sigma-Aldrich, Germany; 100 ng per egg) group, iii) Fadrozole hydrochloride (0.1 mg per egg) + rhIGF-I (100 ng per egg) injected group, iv) a vehicle injected sham control group containing 10 mM acetic acid (#A3686, PanReac AppliChem, Germany) and 0.1% Bovine serum albumin (#A2153, Sigma-Aldrich, Germany), and v) a non-injected control group. Prior to the injections, the shell surface was wiped using 70% ethanol and a small tiny hole was drilled on the surface of the air sack. Injections were made into the albumen right under the air sack using 23-gauge needles cut to 13 mm, then the holes were sealed with liquid paraffin and the eggs were replaced in the incubator in order to be hatched.

### Hatching time and chicks body weight

To determine the period after which the incubated eggs hatched (“hatching time”), the hatched chicks from each group was recorded every 3 hours from 456 to 510 hours of incubation. To follow the standards [23], all the chicks in each group were kept in the hatchery until six hours after the last hatching event and then they were sexed and reared for six weeks. Therefore, we could not determine chicken gender at the exact time of hatching. The hatched chicks were individually weighed and 8 chicks per replicate were randomly distributed into floor pens covered by sawdust as litter (10 birds/m2) and raised for 6 weeks. A corn-soybean diet was formulated to meet or exceed the nutrient requirements of broilers offered by the Ross Broiler Manual (2007) for different periods provided *ad libitum* throughout the experiment.

### Phenotypic and genotypic sex determination

On the day of hatch, the phenotypic sex of randomly selected hatched chicks was evaluated through vent sexing by expert staffs. In summary, following squeezing the vent by fingers and expelling the chick dropping, the vent was opened, and the presence of a bump was considered as the male indicator. Afterwards, these chicks were slaughtered and their liver samples were collected and used to determine their sexual ZZ or ZW genotype. Next, all birds reared for 6 weeks, and the presence or absence of the testis was considered to determine the chicken phenotypic sex following slaughter on Day 42 of production.

Genomic DNA was extracted from the liver samples collected on the hatching day or Day 42 after hatch, and then the genetic sex was identified by PCR from the CHD1 gene [24]. The principle of identifying genetic sex via CHD1 was based on the number of amplified fragments. That is, one or two bands are detectable in genetically male or female chicks, respectively [25].

### Serum biochemical parameters

On Day 1, 2.5 ml of blood was collected by puncturing the heart from four randomly selected birds per replicate (16 chicks per treatment). Blood samples were immediately aliquoted into non-anticoagulant tubes and allowed to clot for 2 hours at 37°C. Then the serum samples were isolated by centrifugation at 2000×g for 10 minutes and stored at −20°C for later analysis. Serum samples were thawed and glucose, total protein, and triglyceride levels were determined using an auto analyzer following the instructions of the corresponding reagent kit (Autolab, PM 4000, Autoanalyzer Medical System, Rome, Italy).

### Statistical analysis

The collected data of hatchability, sexual mortality, and the rate of sex reversal were analyzed by χ2 test. The data obtained from serum biochemical parameters and productive performance attributes of genetically male and female sexes were subjected to the analysis of variance using the general linear model (GLM) procedure in SAS 9.2 package (each sex in isolation). The Tukey post-hoc test was used to assess any significant differences among treatments in each sex. In addition, an independent t-test was used to perform a mean comparison between genetically male and female chicks in each group. Since the frequency distribution of hatching time can be an important factor for the chicken quality dispersion through the duration of early deprivation period, further descriptive statistics including skewness and kurtosis were measured by Microsoft Office Excel 2007. The P-value set at ≤0.05 was considered as the significant level for mean comparisons. All values are presented as mean ± SEM, unless indicated otherwise.

## Results

### Hatching time, hatchability and mortality

**Figure 1:**
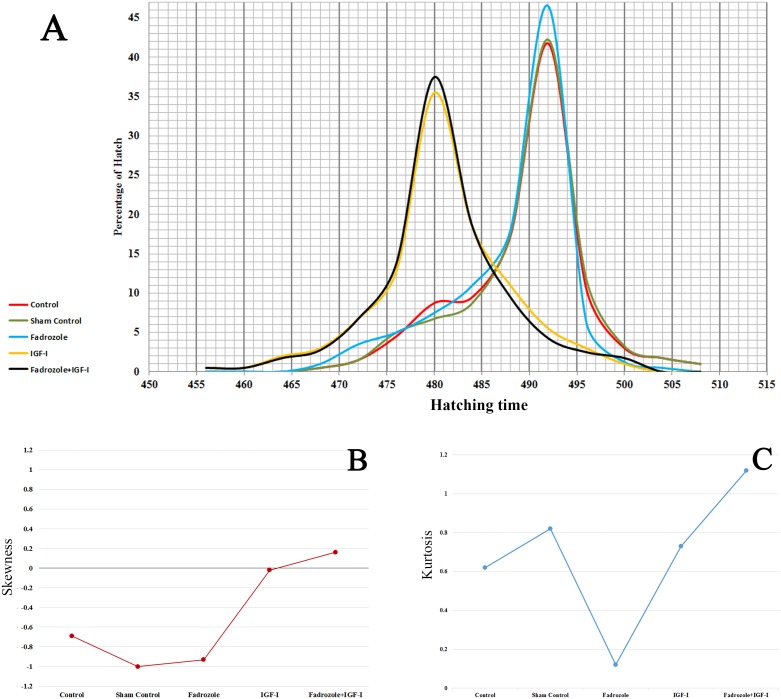
Distribution frequencies of the chicken hatching time. (A) Graphical presentation of hatching time in Control, Sham injection control, Fadrozole (0.1 mg), IGF-I (100 ng), Fadrozole (0.1 mg) + IGF-I (100 ng) groups. All injections were carried out on Day 3 of egg incubation over a long distance from the embryo location. (B) Skewness and (C) kurtosis criteria of the distribution frequencies.

In this study, we evaluated if the administration of Fadrozole and IGF-I, alone or in combination, could affect the hatching traits of broiler chickens. Along with the graphical distribution of hatching time (Figure 1A), we measured skewness (Figure 1B), which demonstrates the amount and direction of departure from horizontal symmetry, and kurtosis (Figure 1C), which reflects the mass movement of hatching time events at peak, tails, and shoulders of the distribution [26]. In the control groups, hatching occurred between 456 and 508 hours post-incubation (Figure 1A) and its distribution was slightly left-skewed (Figure 1.B). Moreover, the mode of hatching time was observed at the 492nd hour of incubation in the control group. Fadrozole noticeably decreased the kurtosis criterion of the frequency distribution (Figure 1.C), without changing the skewed distribution. The mode of hatching time was observed 12 hours earlier in IGF-I injected eggs, irrespective of whether Fadrozole was present or not. However, the complementation of Fadrozole with rhIGF-I slightly increased the kurtosis and hatching events around the average. There were no significant differences in hatchability among experimental groups (Table 1). Embryo mortality was not statistically affected by the treatments, although the number of dead female embryos was higher than males in all experimental groups (Table 1).

**Table 1:** Hatchability and mortality characters of *in ovo* exposed chicken embryos with Fadrozole and IGF-I.

Treatment	Fadrozole (mg)	IGF-I (ng)	No. of eggs	No. of Hatching	Hatchability (%)	No. of Mortality (PCR)	
						♂	♀
Control	0	0	96	76	79.1	8	12
Sham (PBS)	0	0	96	73	76	11	12
Fadrozole	0.1	0	96	74	77.1	8	14
IGF-I	0	100	96	75	78.1	10	11
Fadrozole+IGF-I	0.1	100	96	72	75	9	15

No significant difference was observed at P-value<0.05 among different groups.

### Rate of sex reversal

Following the hatch, a randomly selected sample of chicks from each group underwent phenotypic and genotypic sexing using vent sexing and PCR-based procedures, respectively (Table 2). Even though allocation of fertilized eggs to each group was carried out randomly, a high level of genotypic sex ratio distortion was observed among different groups. Genotypic and phenotypic sexing were in complete agreement for the control, sham injection, and rhIGF-I groups. However, this consistency was not observed for the chicks in either of Fadrozole administrated groups (P≤0.05). Fadrozole injection on Day 3 of incubation led to 89.5% female-to-male sex reversal, while Fadrozole combined with rhIGF-I completely reversed the female phenotype. To see if the female-to-male sex reversal is a stable or transient phenomenon, chickens on 42 days of production from each group were further used for phenotypic and genotypic sexing (Table 3). In agreement with the Day 1, sex-reversal data, 90% of genetically female chicks were masculinized using Fadrozole manipulation on Day 42 of production. Additionally, the synergistic effect of rhIGF-I and Fadrozole, 100% female-to-male sex reversal, was also verified at the end of production period.

**Table 2:** Sexing on the hatch day of Fadrozole/IGF-I treated chicken embryos.

Treatment	Fadrozole (mg)	IGF-I (ng)	No. of Chicks	Vent Sexing		Genotype Sexing		Rate of sex Reversal (%)
				♂	♀	♂	♀	
Control	0	0	44	24	20	24	20	0
Sham (PBS)	0	0	41	28	13	28	13	0
Fadrozole	0.1	0	42	40	2	23	19	90
IGF-I	0	100	43	16	27	16	27	0
Fadrozole+IGF-I	0.1	100	40	40	0	18	22	100

Rate of sex reversal: ratio of number of female-to-male sex-reversed females over number of genetic females at hatch.

**Table 3:** Sexing on 42 days post-hatch of Fadrozole/IGF-I treated chicken embryos.

Treatment	Fadrozole (mg)	IGF-I (ng)	No. of Chicks	Anatomical morphology		Genotype Sexing		Rate of sex Reversal (%)
				♂	♀	♂	♀	
Control	0	0	32	14	18	14	18	0
Sham (PBS)	0	0	32	19	13	19	13	0
Fadrozole	0.1	0	32	31	1	22	10	90
IGF-I	0	100	32	21	11	21	11	0
Fadrozole+IGF-I	0.1	100	32	32	0	13	19	100

Rate of sex reversal: ratio of number of female-to-male sex-reversed females over number of genetic females on Day 42 of production.

### Serum biochemical parameters

The treatment effects on serum constituents which were tested on the day of hatching are summarized in Table 4. Birds from both male and female genotypes which received the rhIGF-I had significantly higher serum protein (g/100 ml), triglyceride (mg/dl) and glucose (mg/dl) concentrations compared to the other groups (P≤0.05). However, no significant difference was observed between genetically male and female chicks in all groups for the evaluated biochemical parameters.

**Table 4:** Serum biochemical parameters of hatched chickens which were exposed to Fadrozole and IGF-I *in ovo*.

Treatment	Fadrozole (mg)	IGF-I(ng)	No. ofChicks	Protein (g/100 ml)*		Triglyceride (mg/dl)*		Glucose (mg/dl)*	
				♂	♀	♂	♀	♂	♀
Control	0	0	16	3.53±0.04**^b^**	3.57±0.06**^b^**	70±8.1**^b^**	66±7.6**^b^**	207±2.7**^b^**	211±3.2**^b^**
Sham (PBS)	0	0	16	3.49±0.06**^b^**	3.40±0.07**^b^**	65±7.8**^b^**	63±7.9**^b^**	210±3.1**^b^**	209±3.3**^b^**
Fadrozole	0.1	0	16	3.45±0.04**^b^**	3.49±0.06**^b^**	69±6.2**^b^**	68±6.8**^b^**	206±3.3**^b^**	203±3.1**^b^**
IGF-I	0	100	16	4.36±0.05**^a^**	4.26±0.07**^a^**	97±4.9**^a^**	96±5.9**^a^**	222±3.7**^a^**	219±3.1**^a^**
Fadrozole+IGF-I	0.1	100	16	4.43±0.05**^a^**	4.39±0.04**^a^**	91±5.7**^a^**	93±5.2**^a^**	217±2.3**^a^**	221±3.2**^a^**
P-Value				0.01	0.01	0.01	0.01	0.006	0.004

*Data were compared using Tukey’s test in the general linear model (GLM) procedure and are shown with mean ± SE.

a,b Different letters at columns denote significant difference (P≤0.05) and same letters at columns denote no significant difference. For all traits, no significant difference was observed between two sexes in each group (P<0.05). P-Value row indicates the P-value of the calculated F statistics for the treatment effect following the analysis of variance.

### Post-hatch productive performance

**Figure 2:**
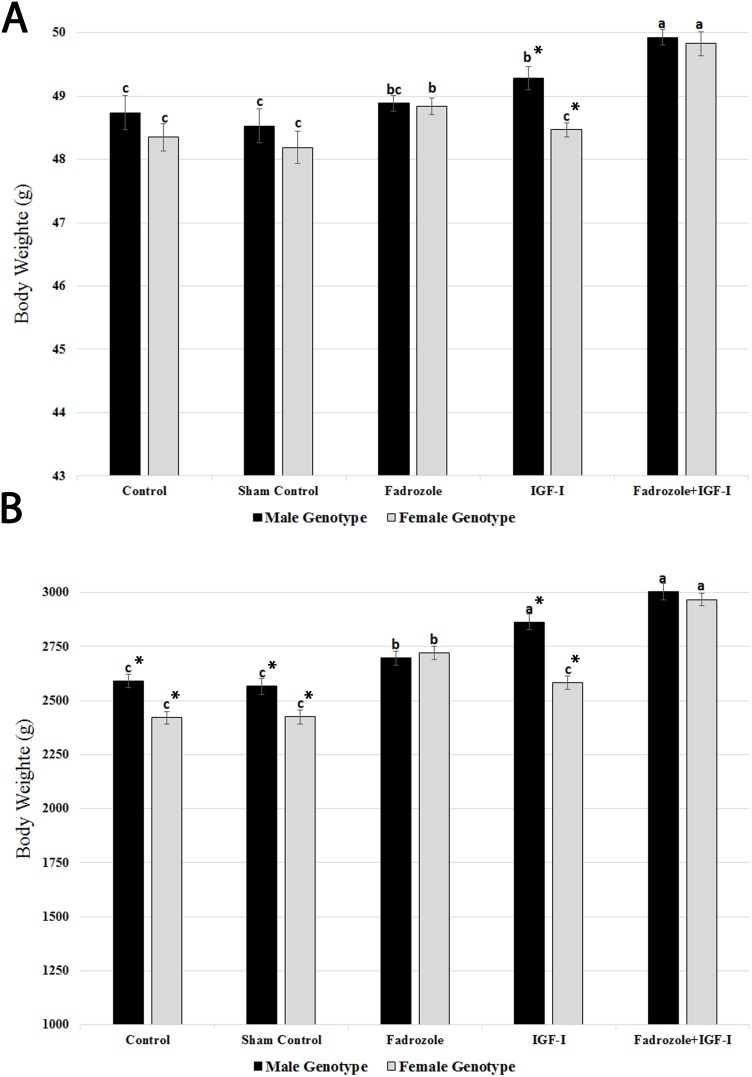
Body weight on Day 1 and 42 of production from *in ovo* exposed chicken embryos with Fadrozole and IGF-I. The impact of chicken embryo treatment on Day 3 of incubation with Fadrozole (0.1 mg) and IGF-I (100 ng) on the average body weight (g) is depicted in (A) 1-day-old and (B) 42-day-old chicks. For the data analysis, chicks were divided into male and female sub-groups, based on ZZ/ZW genotype. Mean comparisons among five groups for each sex were carried out using Tukey post-hoc test in general linear model (GLM) procedure. In addition, mean comparison between male and female sub-groups of each treatment was conducted using an independent t-test, and the significant differences are denoted by an asterisk (*). ^ab^Groups with identical superscripts in each specific gender denote no significant difference (P≥0.05).

The body weight of genetically male and female chicks on Day 1 and 42 of production are presented in Figure 2. Initial body weight at Day 1 was not significantly different between males and females in the control groups (Figure 2A). Fadrozole injection increased the weight of females, with slight impact on the male body weight. On the other hand, IGF-I supplementation considerably improved the male weight gain without changing the female average weight. This resulted in significant difference between male and female body weight in this group. The combined application of Fadrozole and IGF-I further improved the Day 1 body weight of both males and females compared to other groups. Following up the body weight till Day 42 of production (Figure 2B) indicated that unlike Day 1 weight records, there were significant different between male and female chicks in both non-injected and sham injection control groups. At this stage, Fadrozole increased the average weight of male chicks as much as that of the female chicks. This was the only group in which females showed numerically higher weight compared to the male chicks. In accordance with the Day 1 records, IGF-I significantly affected only the male chicks, with no considerable effect on females. Thus, Fadrozole attenuated the weight differences between the two genders, while IGF-I intensified the male weight gain. In accordance with the Day 1 data, simultaneous administration of Fadrozole and IGF-I increased Day 42 body weight of both males and females equally.

## Discussion

In the current study, chicken embryos were treated with recombinant human IGF-I and Fadrozole, as a nonsteroidal anti-aromatase reagent, on Day 3 of incubation. We evaluated the simultaneous and separate effects of these two compounds on female-to-male sex reversal and body weight on the Day 1 and 42 of production as well as the hatching traits. Strong similarity of human and chicken IGF-I protein sequence [27], [28], very high affinity of rhIGF-I to the chicken IGF-I receptors [29], and successful usage of rhIGF-I for improving chicken body weight [16], [22] persuaded us to use rhIGF-I in the current study. We showed that IGF-I combination with Fadrozole could enhance female-to-male sex reversal, shorten hatching time, and increase chicken body weight.

### Female-to-male sex-reversal

Sex-reversal was the main agenda of this project. Determination of chicken gender was carried out based on vent phenotype and ZW genotype on the first day of production. Based on phenotype and genotype sexing being in complete agreement, neither sham injection nor rhIGF treatment induced sex-reversal. By contrast, Fadrozole effectively converted females to male chicks with 90% efficiency. The obtained results for Day 1 was consistent with previous reports on aromatase-inhibitors [30], [31]. Aromatase, the key enzyme responsible for estrogen synthesis [32], expresses specifically in the female chicken gonads on the Day 5 of incubation, the time of gonadal sex differentiation [32], [33]. Thus, estrogen synthesis during chicken embryonic stages is restricted to female [32], although the expression of estrogen receptors commences prior to the gonadal differentiation, on Day 4.5 in both male and female chicks [4], [32], [33]. The difference in expression pattern of P450 AROM, CYP19 gene, could be attributed to alternate active promoters in different tissues [34]. Recently, it has been shown that aromatase over-expression prior to sex differentiation can induce male-to-female chicken sex reversal [1]. We further evaluated the post-hatch stability of the sex-reversal and repeated our early results on Day 42 of production. Stability of chicken sex-reversal by anti-aromatases has been controversial among researchers and was suggested to be anti-aromatase dose-dependent [30], [31]. However, we found that using low dosage of an anti-aromatase, irreversible female-to-male sex-reversal could be achieved. This discrepancy might partly be due to the differences in the quality and activity of Fadrozole produced by different companies.

Even though IGF-I did not lead to sex-reversal in this study, 100% of genetically female chicks associated with male characteristics when a combination of IGF-I and Fadrozole was injected *in ovo*. The masculine phenotypes were permanently observed in the genetically female chicks through 42 days of production. Application of growth hormones (GH) improved spermatogenesis, serum testosterone, estradiol and serum IGF-I [35]. Further studies clearly proved that the GH mechanism of action is mediated through IGF-I synthesis [36]. Following IGF-I treatment of humans and mice suffering from primary growth hormone resistance, serum IGF-I level increased drastically and led to a substantial increase in serum LH, FSH and testicular testosterone [37], [38]. Moreover, polymorphisms in androgen receptor [39], estrogen receptors [40] and aromatase [39], [40] genes have been shown effective on human sperm concentration and motility. In addition to FSH and LH, IGF-I is thus an essential requirement for sexual development and testicular function (see [41] for a review). The mechanism of action for IGF-I effects on the reproduction system has not been fully understood but could involve regulation of the aromatization process [19], [42]. The administration of IGF-I reduced aromatase gene expression in Sertoli cells through interfering with either the production or the degradation of mRNAs [19]. It had similar effects to FSH in terms of increasing cellular proliferation, lactate production as well as glucose and amino acid transfer [43]. FSH can quickly down-regulate the IGF-I binding protein 3 (IGFBP-3) gene expression and increase IGF-I activity [43]. Moreover, the IGF-I inhibitory effect of IGFBP-3 is dose-dependent and can be surpassed by higher concentration of IGF-I [43]. This anti-aromatase activity of IGF-I in Sertoli cells is in contrast to enhancing aromatase activity in granulosa cells [42]. IGF-I treatment of human and mouse granulosa cells increased estradiol synthesis [20], [44], [45] and P450AROM activity [46]. It has also been confirmed that the IGFBP-3 action mechanism could attenuate FSH-stimulated estrogen and progesterone in the granulosa cell [47]. Moreover, changes in androstenedione levels following IGF-I manipulation also differed in females versus males [37], [48]. Chicken gonads are uncommitted until Day 4.5, retaining the potential to be either ovary or testis. If the aromatase-stimulating activity of IGF-I takes place in chicken granulosa cells [1], it should interfere with the anti-aromatase activity of Fadrozole treatment and subsequently decreases the female-to-male sex reversal chance. However, the female-to-male sex-reversal event was favored by the simultaneous treatment of IGF-I and the anti-aromatase agent in this study.

### Chicken body weight

We further investigated if the sex-reversal event is associated with the chicken body weight gain. Fadrozole significantly increased the initial body weight of genetically female chicks. Estrogen enhances skeletal muscle catabolism, while testosterone increases skeletal muscle anabolism [49]. The positive effects of androgens on muscle mass could be mediated through IGF-I expression in avian muscles [50]. It is noteworthy that in our study Fadrozole also significantly increased the post-hatch body weight of genetically male chicks. To our knowledge, this is the first report on stimulating the male chicken weight gain by using an anti-aromatase agent. Fadrozole-treated embryos, with or without IGF-I, were the only group in which no significant difference was observed between genetically male and female chicks by the end of Day 42 of the production period. Even though lack of expression [32], [33] or very weak expression [7], [51] of the aromatase gene was observed in the early embryonic stages of male chick gonads, its considerable expression was recorded in the chicken matured testis [33] and human early prepubertal testis [52]. Therefore, the anti-aromatase compounds are less likely to be helpful during sex differentiation, and possible auxiliary effects probably occur during the post-differentiation period after Day 9.5. We injected Fadrozole and IGF-I compounds over a wide region in the embryo position, rather than in its adjacent area. This should have decreased the bulk access and uptake of these reagents at the sexual differentiation stage and extend their availability in the chicken egg media. With respect to the latter, a series of protease inhibitors, including ovomucoid [53], ovostatin [54] or ovomacroglubulin [55], ovoinhibitors [53], [56], and chicken cystatin (Barrett, 1981), exist in chicken egg albumen [see [57] for review]. These protease inhibitors can strongly inhibit various types of proteases [see [57] for review], protect the injected proteins from degradation assays and extend their half-life.

IGF-I substantially increased male chick body weight at Day 1 and subsequently Day 42 of production. IGF-I plays a crucial role in the growth and development during embryonic period as well as post-hatch period [58]. Over the past years, the role of IGF-I as a potent mitogen which can stimulate satellite cell activation has also been illustrated [59]–[61]. IGF-I receptors are distributed in various organs, including brain, pituitary, gonads, and reproductive tracts [41]. This suggests that every component of hypothalamic-pituitary-gonad axis could be the action site for IGF-I through endocrine and paracrine systems [41]. However, in agreement with previous publications [16], [21], [22], IGF-I impact was restricted to the male chicks. This indicates the presence of very strong interaction between sexual hormones and IGF-I. Estrogen synthesis is the key difference between chicken genders [32], [33]. If estrogen blocks the IGF-I receptors or induces the IGFBP-3, inhibiting estrogen synthesis by aromatase inhibitors should de-escalate the insensibility of genetically female embryos to IGF-I protein. Interestingly, results of this study clearly showed that this hypothesis could be true and genetically female embryos, which received both Fadrozole and IGF-I, had significantly higher body weight compared to those which received either IGF-I or Fadrozole. Despite intense research in other areas, such as the nervous system [62] and cancer [63]–[65], the exact IGF-I/estrogen interaction underlying weight gain has not been fully elucidated. In summary, synergistic effects of Fadrozole and IGF-I on sex-reversal and weight gain of broiler chicks, could be promising for further improving the broiler chicks industry, as well as finding new therapeutic systems in assisted reproductive technologies (ART) [66] or growth disorders.

### Hatching characters

Along with the sex-reversal and weight gain of chicks, we evaluated if the IGF-I and Fadrozole effects can influence the hatching traits, as important factors in the chicken hatchery section. Our results confirm that hatchability and mortality traits were not affected by the treatment groups. However, application of higher doses of Fadrozole led to remarkable decrease in hatchability [10], [11], [31], [67]. The non-significant effect of treatment groups on mortality and hatchability might be partly due to the injection site. Genotypic sexing of dead embryos revealed that the number of dead female embryos was higher than that of the male counterparts. This finding confirms previous reports of higher early death in female embryos in some bird species [13], [68]. Furthermore, the sex-reversed groups, similar to other groups, had a higher mortality rate of genetically female compared to male embryos. These observations may further illustrate that the difference in early embryonic survival rates of females and males are likely due to the difference in the number of alleles for genes which are located on the Z chromosome but are not involved in the sexual differentiation.

Results from each 3-hour inspection period of hatchery machine showed that the hatching time was left-skewed and indicated abundance of late hatchers in the control groups. Supplementation of Fadrozole into chicken eggs increased the number of hatched chicks around the mode and afterwards increased the number of late hatchers. The spread of hatching time can be escalated up to 48 hours for late versus early hatchers [23]. Therefore, practically, chicks are deprived from water and feed for up to 72 hours post-hatching [69]. On the other hand, early hatchers suffer more from being fasted compared to the late hatchers [23], have slower growth rate and immune system activity [69]–[71], and comprised the majority of low quality chickens [70]. Moreover, the compulsory holdup feed access is a main aggravating factor for long storage duration of hatched chicks [70]. Therefore, increasing the number of late hatchers can attenuate detrimental effects of the early deprivation period.

Inclusion of recombinant human IGF-I into chicken egg albumen, with or without Fadrozole, noticeably advanced the hatching time. Shortening the hatching window through setting and hatching period was suggested as an important pathway to attenuate possible detrimental effects of the quite long early deprivation period [69]. The effects of IGF-I on shortening hatching time could be attributed to accelerating embryo development [23] and subsequently body weight gain of chicken embryos. Since we needed to wait until all the eggs hatched and then removed them from the incubator [23], we could not differentiate the gender of chickens at the exact hatching time. However, this could be worthy note to distinguish the gender effect at the hatching time and assess the possible associations between the gender, body weight and hatching time variables. Additionally, the IGF-I and Fadrozole combination improved the symmetrical shape of the distribution by decreasing the departure from normality and slightly increased the mass of hatches around the average. The more uniform hatching events through IGF-I plus Fadrozole treatment were associated with the less dispersed body weight at Day 1 of production. This could be of high importance in broiler chicken industry, since the spread of the hatching time and the maintenance condition following the hatch in the hatchery machine is a crucial step in the production of Day 1 old chicks [72]. In the current study, application of IGF-I substantially advanced the hatching time and improved the spread of hatches. In addition, comparison of serum biochemical parameters showed that chicks received IGF-I had higher serum glucose and total protein and fatty acids compared to other groups. These could be indicated as higher metabolic rate, available energy sources and healthy parameters [69] in the IGF-I treated chicks. Although lower plasma glucose and T3 was observed in chicks from early hatching period [23], IGF-1 treated chicks hatched earlier and showed significantly higher serum biochemical parameters in the current study. Taken together, *in ovo* treatment of chicken embryos with IGF-I alleviated the newly hatched chick’s early feeding problems in several ways, including shortened and normalized hatching time, increased spread of hatch around the average, and enhanced serum biochemical parameters of the hatchers.
